# Single Cell and Bulk RNA-Seq Profiling of Non-Metastatic Versus Bone-Metastatic Prostate Cancer Identifies the CXCL10-CXCR3 Axis as a Key Determinant of Tumor Microenvironment and Treatment Resistance

**DOI:** 10.3390/biomedicines14040943

**Published:** 2026-04-21

**Authors:** Zijian Song, Likai Ren, Hong Wang, Yanqing Wang, Xinxing Du, Wei Zhou, Qi Zhang, Jiyuan Yu, Zaixu Zhao, Linxiong Ye, Kaidi Jin, Ying Liu, Wei Xue

**Affiliations:** 1 Department of Urology, Renji Hospital, Shanghai Jiao Tong University School of Medicine, Shanghai 200127, China; drsongzj@163.com (Z.S.); renlkdoctor@163.com (L.R.); wanghong@renji.com (H.W.); iwangyq@163.com (Y.W.); dxx9607@163.com (X.D.); 21239@renji.com (W.Z.); zhangqi6212023@126.com (Q.Z.); yujiyuanq@126.com (J.Y.); 2 Department of Biological Medicines & Shanghai Engineering Research Center of Immunotherapeutics, School of Pharmacy, Fudan University, Shanghai 201203, China; 13053769593@163.com (Z.Z.); yelinxiong@gmail.com (L.Y.); 3 Department of Forensic Medicine, School of Basic Medical Sciences, Fudan University, Shanghai 201203, China; jinkd@fudan.edu.cn; 4 Jiangsu Provincial Engineering Research Center of Cancer Cell Therapy and Translational Medicine, Xuzhou City Engineering Research Center of Cancer Cell Therapy and Translational Medicine, Xuzhou Central Hospital, Southeast University, Xuzhou 221009, China; 5 School of Life Sciences, Jiangsu Normal University, Xuzhou 221116, China

**Keywords:** bone metastasis, bulk RNA sequencing, CXCL10-CXCR3 axis, prostate cancer, single-cell sequencing, tumor microenvironment

## Abstract

**Background:** Bone metastasis is a major determinant of morbidity and therapeutic failure in advanced prostate cancer (PCa); however, the transcriptional programs and tumor microenvironmental alterations driving metastatic progression remain incompletely understood. This study aimed to systematically characterize transcriptomic differences between non-metastatic and bone-metastatic PCa and to identify key microenvironmental signaling pathways involved in tumor survival and chemoresistance. **Methods:** Bulk RNA sequencing was performed on 49 non-metastatic and 28 bone-metastatic PCa specimens. Differential expression analysis was integrated with weighted gene co-expression network analysis (WGCNA), gene set enrichment analysis, and immune/stromal deconvolution. Key findings were validated using in vitro functional assays, including Transwell co-culture models, small interfering RNA (siRNA)-mediated gene silencing, cell viability, apoptosis, and docetaxel resistance analyses. **Results:** Transcriptomic profiling identified 574 differentially expressed genes. Bone-metastatic tumors were enriched in ribosome-related and translational pathways, whereas non-metastatic tumors displayed immune-associated signatures, including natural killer (NK) cell-mediated cytotoxicity and cytokine signaling. WGCNA revealed immune-related gene modules preferentially enriched in non-metastatic disease. Immune deconvolution demonstrated significantly higher infiltration of NK cells and endothelial cells in non-metastatic tumors. Chemokine-receptor analysis highlighted upregulation of the CXCL10-CXCR3 axis in non-metastatic PCa. In vitro, PCa cells expressed CXCR3, while endothelial cells markedly increased CXCL10 expression upon co-culture. Functional assays showed that endothelial-derived CXCL10 promoted PCa cell survival, suppressed apoptosis, and conferred resistance to docetaxel via CXCR3-dependent signaling; these effects were reversed by CXCL10 or CXCR3 knockdown. **Conclusions:** These findings uncover a context-dependent endothelial-immune chemokine network distinguishing non-metastatic from bone-metastatic PCa and identify the CXCL10-CXCR3 axis as a critical mediator of tumor survival and chemoresistance, suggesting a potential therapeutic vulnerability in advanced prostate cancer.

## 1. Introduction

Prostate cancer (PCa) remains the most commonly diagnosed solid malignancy among men and a leading cause of cancer-related morbidity and mortality globally [1]. Its clinical behavior is remarkably heterogeneous, spanning indolent localized tumors to highly aggressive disease with early dissemination. Although substantial advances in screening, molecular classification, and primary treatment have improved outcomes for many patients, metastatic progression continues to represent the critical inflection point leading to incurable disease [2]. Indeed, the survival of individuals with advanced PCa is largely dictated by the onset and extent of metastatic spread [3].

Among metastatic sites, bone is the overwhelmingly predominant destination, affecting approximately 80–90% of patients with advanced or castration-resistant prostate cancer [4]. These lesions are predominantly osteoblastic but often coexist with osteolytic components, leading to a complex remodeling of the bone microenvironment. Bone metastases are responsible not only for debilitating skeletal-related events—such as fractures, spinal cord compression, intractable bone pain, and hypercalcemia—but also for substantially shortened survival [5,6]. The affinity of PCa cells for the bone microenvironment is thought to reflect a convergence of tumor-intrinsic properties and the unique biological landscape of bone, which includes abundant growth factors, cytokines, mineralized matrix components, and a dynamic interplay between osteoblasts, osteoclasts, endothelial cells, and immune cells [7].

The establishment and expansion of PCa bone metastases involve a complex, multistep cascade encompassing tumor cell detachment, intravasation, survival in circulation, extravasation into bone, and colonization of the marrow niche [8]. Once in the bone microenvironment, disseminated tumor cells engage in reciprocal interactions with stromal elements, initiating a “vicious cycle” wherein tumor- and stroma-derived factors cooperatively drive osteoblastic and osteolytic remodeling, thereby supporting tumor proliferation and metastatic outgrowth [9]. Interactions between metastatic tumor cells and stromal components alter the delicate balance of bone formation and resorption, creating a “vicious cycle” that further promotes tumor growth and osteogenic activity [10]. Despite substantial progress in understanding the metastatic cascade, the precise mechanisms that enable PCa cells to colonize bone and reshape its microenvironment remain incompletely elucidated.

Importantly, the bone microenvironment is not a passive bystander but an active participant in metastatic progression. Stromal and immune components including endothelial cells, myeloid cells, osteoclast precursors, and lymphocyte subsets, play critical roles in shaping metastatic niches [11,12]. The immune contexture of PCa is notably distinct from immunologically “hot” tumors; PCa often exhibits sparse effector immune infiltration, dysfunctional innate responses, and limited responsiveness to immunotherapy [13]. These observations underscore the need for deeper characterization of microenvironmental differences between localized and bone-metastatic PCa, particularly regarding immune composition, stromal activation, and transcriptional reprogramming.

Recent transcriptomic studies have begun to illuminate the substantial heterogeneity across PCa subtypes and metastatic states, revealing alterations in immune signaling, metabolic pathways, translational regulation, and stromal remodeling [14,15]. However, integrative analyses that directly compare localized and bone-metastatic tumors remain limited, especially within clinically annotated patient cohorts. A clearer understanding of the microenvironmental reprogramming that accompanies the transition from localized PCa to bone metastatic disease may uncover biological determinants of metastatic competence and identify potential therapeutic vulnerabilities.

To address this knowledge gap, we conducted a systematic analysis of tumor tissues from both non-metastatic and bone-metastatic PCa patients to delineate the transcriptional, cellular, and microenvironmental alterations that accompany bone metastatic progression. By integrating bulk RNA sequencing with comprehensive histopathologic and microenvironmental profiling, our study seeks to define the molecular programs that distinguish bone-metastatic from localized disease and to identify context-specific changes within immune and stromal compartments that contribute to metastatic dissemination.

## 2. Methods

### 2.1. Sample Collection

This study was designed as a retrospective cohort analysis. A total of 77 prostate cancer (PCa) patients who underwent radical prostatectomy at the Shanghai Renji Hospital were included, comprising 49 patients without bone metastasis (non-metastatic group) and 28 patients with bone metastasis (bone-metastatic group). Bone metastatic status was determined based on radiologic and clinical evaluation at the time of diagnosis. All 77 patients were treatment-naïve at the time of tissue acquisition, with no prior history of androgen deprivation therapy (ADT), chemotherapy, or radiotherapy. This criterion was strictly applied to avoid treatment-related transcriptomic alterations and to capture baseline molecular signatures. Importantly, all tissue samples analyzed in this study were derived from primary prostate tumors obtained during radical prostatectomy. No metastatic bone lesion samples were included. For patients in the non-metastatic group, radical prostatectomy was performed as the standard primary treatment for localized disease. For patients in the bone-metastatic group, radical prostatectomy was conducted in carefully selected clinical contexts, such as oligometastatic disease, cytoreductive intent, or symptom control, based on multidisciplinary team evaluation and institutional clinical practice. Thus, the comparison in this study was performed between two independent patient cohorts: primary prostate tumors from patients without bone metastasis and primary prostate tumors from patients with bone metastasis. No matched primary and metastatic tissue pairs from the same patients were available. Detailed clinicopathological characteristics of the 77 patients are summarized in Table 1 and Supplementary Table S1.

Immediately after surgical resection, tumor tissues were divided into two portions: one part was snap-frozen in liquid nitrogen and stored at −80 °C for RNA extraction, and the other part was formalin-fixed and paraffin-embedded (FFPE). Hematoxylin and eosin-stained sections were reviewed by a pathologist to confirm tumor content, and samples with less than 70% tumor cellularity were macrodissected prior to downstream analyses.

This study was conducted in accordance with the Declaration of Helsinki and approved by the Ethics Committee of Renji Hospital Affiliated with Shanghai Jiao Tong University School of Medicine. Written informed consent was obtained from all participants.

Immediately after surgical excision or biopsy, fresh tumor specimens were divided into two portions: (i) one part was snap-frozen in liquid nitrogen and stored at −80 °C for RNA extraction and sequencing, and (ii) the remaining part was fixed in 10% neutral-buffered formalin and embedded in paraffin (FFPE) for RNA-seq. Due to the superior RNA stability and archival feasibility for clinical specimens, all RNA sequencing in this study was performed using FFPE tissues. Hematoxylin and eosin-stained sections were reviewed to confirm tumor content, and samples containing <70% tumor cells were macrodissected to enrich tumor areas prior to downstream analyses.

This study was conducted in accordance with the Declaration of Helsinki and approved by the Ethics Committees of Renji Hospital Affiliated with Shanghai Jiao Tong University School of Medicine. Written informed consent was obtained from all participants prior to tissue collection and data usage.

### 2.2. RNA Extraction and Bulk RNA Sequencing

Total RNA was extracted from FFPE prostate cancer tissue sections (5–10 μm) using the RNAprep Pure FFPE Kit (Tiangen, Beijing, China) following the manufacturer’s instructions. Tissues were deparaffinized with xylene, washed with ethanol, and air-dried. After lysis and Proteinase K digestion at 56 °C, crosslink reversal was performed at 80 °C. RNA was purified via column-based adsorption, eluted in RNase-free water. RNA quality was assessed using the Agilent 2100 Bioanalyzer (Santa Clara, CA, USA), and only samples with DV200 values > 30% (percentage of RNA fragments > 200 nucleotides) were selected for library construction and subsequent sequencing. RNA quantification was performed using a NanoDrop 2000 spectrophotometer (Waltham, MA, USA). RNA integrity was assessed with the Agilent 2100 Bioanalyzer; only samples meeting quality thresholds for FFPE-derived sequencing were selected for library construction.

RNA sequencing libraries were prepared using the Illumina TruSeq Stranded mRNA Library Prep Kit (Illumina, San Diego, CA, USA). Poly(A)+ mRNA was enriched, fragmented, and reverse-transcribed into cDNA. After end repair, A-tailing, and amplification, libraries were quantified with Qubit and pooled. Paired-end sequencing (150 bp) was performed on an Illumina NovaSeq 6000 platform. Raw reads were quality-checked with FastQC, trimmed with Trimmomatic, and aligned to the GRCh38 human genome using STAR. Gene-level counts were obtained with featureCounts. Differential expression analysis was performed using DESeq2, with genes showing |log2FC| > 1.5 and adjusted *p*-value < 0.05 defined as significant and used in downstream analyses.

### 2.3. Gene Set Enrichment Analysis (GSEA)

GSEA was performed using the clusterProfiler R package (v4.6.0) to identify biological pathways associated with metastatic status. All genes were ranked according to the log2 fold change values derived from DESeq2 differential expression analysis. Enrichment analysis was conducted against the Kyoto Encyclopedia of Genes and Genomes (KEGG) pathway database and Hallmark gene sets obtained from the MSigDB database. A permutation-based approach was applied with 1000 permutations, and enrichment significance was evaluated using normalized enrichment scores (NES). Pathways with a adjusted *p* value < 0.05 were considered significantly enriched.

### 2.4. Immune and Stromal Cell Profiling Using MCPcounter

Immune and stromal cell abundances were estimated using the MCPcounter R package (v1.2.0). Normalized gene expression matrices derived from RNA-seq data were used as input, and cell-type-specific scores were computed based on predefined transcriptional signatures. MCPcounter provides quantitative estimates for major immune and stromal populations, including T cells, endothelial cells, myeloid cells, NK cells, and fibroblasts. The resulting scores were compared between non-metastatic and bone-metastatic PCa samples and incorporated into downstream analyses to evaluate microenvironmental differences associated with metastatic progression.

### 2.5. Weighted Gene Co-Expression Network Analysis (WGCNA)

A weighted gene co-expression network was constructed using the WGCNA R package (v1.72) to identify modules correlated with metastasis and tumor microenvironment features. After filtering low-expression and low-variance genes, a signed network was built by selecting an optimal soft-thresholding power that met scale-free topology criteria. Following the generation of adjacency and topological overlap matrices, modules were identified through hierarchical clustering and represented by module eigengenes. Modules significantly associated with clinical traits or immune/stromal cell infiltration were selected for GO and KEGG enrichment analysis.

### 2.6. GO Enrichment Analysis

Functional enrichment of differentially expressed genes and WGCNA modules was performed using the clusterProfiler R package (v4.6.0). A hypergeometric test was applied with Benjamini-Hochberg correction. Gene Ontology biological process terms with an adjusted *p*-value < 0.05 were considered significantly enriched. Results were visualized using dot plots and enrichment maps to highlight key biological themes related to metastatic progression.

### 2.7. TCGA-PRAD Transcriptomic Analyses

Correlation and immune infiltration analyses were performed using the TIMER platform. Specifically, the association between CXCL10 expression and tumor purity was assessed, and the correlation between CXCL10 and CXCR3 was evaluated using purity-adjusted partial Spearman correlation. In addition, correlations between CXCL10 and immune-related scores as well as NK cell-related infiltration/activation metrics were examined. To investigate CXCL10-associated biological functions, samples were stratified into high, intermediate, and low CXCL10 expression groups, and GSEA was conducted by comparing the high versus low groups. Enrichment analyses were performed for KEGG pathways and GO Biological Process terms, with statistical significance determined by multiple-testing adjusted FDR (*p*.adjust).

### 2.8. Cell Culture and Co-Culture Conditions

Human prostate cancer cell lines PC-3 and LNCaP, along with the human endothelial cell line hCMEC/D3, were obtained from BeNa Culture Collection (Beijing, China). PC-3 is an androgen receptor (AR)-negative, castration-resistant cell line derived from a bone metastasis, while LNCaP is an AR-positive, hormone-sensitive cell line derived from a lymph node metastasis. This pair was selected to represent distinct disease stages and molecular contexts of prostate cancer. PC-3 and LNCaP cells were cultured in RPMI-1640 (Gibco, Grand Island, NY, USA) supplemented with 10% FBS (Gibco, USA), while hCMEC/D3 cells were maintained in EBM-2 medium (Gibco, USA) with 10% FBS and endothelial cell growth supplement. All cells were incubated at 37 °C with 5% CO_2_.

For co-culture experiments, a Transwell system (0.4-μm pore) was used. hCMEC/D3 cells were seeded in the upper chamber and PC-3 or LNCaP cells in the lower chamber (2 × 10^4^ cells/well). Co-cultures and monoculture controls were maintained for 24–72 h as specified.

### 2.9. Quantitative Real-Time PCR (qRT-PCR)

Total RNA was extracted using TRIzol reagent (Invitrogen, Carlsbad, CA, USA) and quantified with a NanoDrop spectrophotometer. cDNA was synthesized from 1 μg RNA, and qPCR was performed with SYBR Green Master Mix (Takara, Shiga, Japan) on a QuantStudio system. Gene expression was normalized to Actin using the 2^−ΔΔCt^ method. All reactions were performed in triplicate.

### 2.10. Enzyme-Linked Immunosorbent Assay (ELISA)

We measured the concentrations of CXCL10 and CXCR3 in the medium supernatant according to the guidelines provided by the reagent supplier. The ELISA kit was purchased from JONLNBIO Co., Ltd. (Shanghai, China). CXCL10 ELISA kit for JL11028, and CXCR3 ELISA kit for JL12547.

### 2.11. Cell Viability Assay

PC-3 and LNCaP cells were plated in 96-well plates and treated with the CXCR3 inhibitor AMG 487 or the agonist PS372424 hydrochloride at varying concentrations for 24, 48, and 72 h. Viability was measured using the CCK-8 assay and all experiments were performed in triplicate. Following reagent addition and incubation at 37 °C, absorbance at 450 nm was recorded. Viability was calculated as a percentage relative to vehicle-treated controls.

### 2.12. Invasion Assay

Cell invasion was assessed using Matrigel-coated 24-well Transwell inserts (8 μm pores). PC-3 and LNCaP cells (1 × 10^5^) in serum-free medium containing the specified treatment were seeded in the upper chamber. The lower chamber contained 10% FBS medium with the corresponding treatment as a chemoattractant. After 24 h, non-invading cells were removed, and cells on the lower membrane were fixed, stained with crystal violet, and counted in five random fields per insert under a light microscope.

### 2.13. Cell Migration Assay

A scratch wound assay was performed on confluent PC-3 and LNCaP monolayers in 6-well plates. A uniform scratch was created with a sterile pipette tip. After washing, fresh medium containing the treatment was added. Wound images were captured at 0 and 24 h using an inverted microscope. Wound closure was quantified as the percentage reduction in wound area at 24 h relative to time zero using ImageJ software (version 2.1.4.7).

### 2.14. Chemoresistance Assay

Cells were pretreated for 24 h with AMG 487, PS372424 hydrochloride (0.1–10 μM), or vehicle (DMSO). The medium was then replaced with fresh medium containing the same modulator and a range of docetaxel concentrations (0–10 nM). After 48 h, cell viability was assessed by the CCK-8 assay. Absorbance at 450 nm was measured, background subtracted, and viability expressed relative to the vehicle-only control. Dose-response curves were generated by plotting viability against log-transformed docetaxel concentrations, and IC_50_ values were calculated using nonlinear regression analysis with a four-parameter logistic (4PL) curve-fitting model in GraphPad Prism (v9.0). For each experimental condition, IC_50_ values were reported together with their 95% confidence intervals to indicate the precision of the estimates.

### 2.15. Small Interfering RNA (siRNA) Transfection

siRNAs targeting human CXCR3 (si-CXCR3), CXCL10 (si-CXCL10), and a non-targeting control (si-NC) were synthesized by GenePharma (Shanghai, China). The sequences used were as follows: si-CXCR3 (sense: 5′-GGAUUAUCCUGUCAUUCUUTT-3′); si-CXCL10 (sense: 5′-GCUUCAGCUUGUGAUCUUCTT-3′); si-NC (sense: 5′-UUCUCCGAACGUGUCACGUTT-3′).

PC-3 and LNCaP cells were transfected at 60–70% confluency using Lipofectamine 3000 (Invitrogen, Carlsbad, CA, USA) according to the manufacturer’s protocol. Briefly, siRNA-Lipofectamine 3000 complexes were formed in Opti-MEM and added to cells at a final siRNA concentration of 50 nM. Cells were harvested 48 h post-transfection for knockdown validation by qRT-PCR.

### 2.16. Flow Cytometric Analysis of Apoptosis

Apoptosis was assessed using an Annexin V-FITC/PI Apoptosis Detection Kit (BD Biosciences, San Jose, CA, USA). Treated cells were collected, washed with PBS, and resuspended in binding buffer. After staining with Annexin V-FITC and PI in the dark for 15 min, samples were analyzed on a BD FACSCanto II flow cytometer (San Jose, CA, USA). After staining, 400 μL of binding buffer was added to each sample, and cells were analyzed within 1 h on a BD FACSCanto II flow cytometer. The gating strategy was as follows: first, cells were gated on a forward scatter-area (FSC-A) versus side scatter-area (SSC-A) plot to exclude cellular debris and aggregates. Next, doublets were excluded using FSC-height (FSC-H) versus FSC-A gating to select single cells. From the single-cell population, apoptotic cells were identified on an Annexin V-FITC versus PI plot. Quadrant gates were set based on unstained controls and single-stained compensation controls. Early apoptotic cells were defined as Annexin V-positive/PI-negative, late apoptotic cells as Annexin V-positive/PI-positive, and necrotic cells as Annexin V-negative/PI-positive. Total apoptotic cells were calculated as the sum of early and late apoptotic populations. Data were processed with FlowJo software(version 10), with apoptotic cells defined as Annexin V-positive.

### 2.17. Statistical Analysis

Statistical analyses were performed using R (v4.2.2) and GraphPad Prism (v9.0). Data are presented as mean ± SD. Two-group comparisons used Student’s *t*-test; multiple-group comparisons used ANOVA with appropriate post hoc tests. Correlations were evaluated by Pearson’s coefficient. For transcriptomic data, *p*-values were adjusted using the Benjamini-Hochberg method. A two-sided *p* < 0.05 was considered significant. Significance levels are indicated as * *p* < 0.05, ** *p* < 0.01, *** *p* < 0.001, **** *p* < 0.0001.

## 3. Results

### 3.1. Transcriptomic Profiling Reveals Distinct Molecular Signatures Between Non-Metastatic and Bone-Metastatic Prostate Cancer

**Figure 1:**
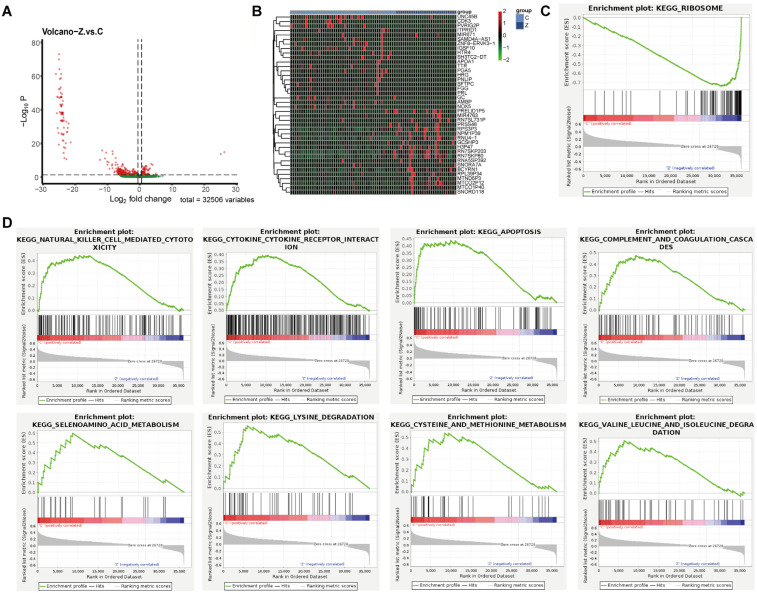
Transcriptomic profiling reveals distinct molecular signatures between non-metastatic and bone-metastatic PCa. (**A**) Volcano plot showing differentially expressed genes between non-metastatic and bone-metastatic PCa samples. (**B**) Heatmap of the top 40 differentially expressed genes, demonstrating clear transcriptional separation between non-metastatic and bone-metastatic tumors. (**C**) GSEA of differentially expressed genes in bone-metastatic PCa. (**D**) GSEA of differentially expressed genes in non-metastatic PCa.

To characterize transcriptional differences between non-metastatic and bone-metastatic PCa, differential expression analysis was performed. In total, 574 DEGs were identified, including 97 upregulated and 477 downregulated genes (Figure 1A). A heatmap of the top 40 DEGs demonstrated clear separation between the two groups, indicating distinct transcriptional states associated with metastatic progression (Figure 1B). To further elucidate the biological processes associated with these transcriptional alterations, GSEA was conducted using ranked gene expression profiles. The results demonstrated that bone-metastatic PCa was significantly enriched for ribosome-related KEGG pathways, suggesting enhanced translational activity in metastatic tumors (Figure 1C). In contrast, non-metastatic tumors were enriched for pathways associated with NK cell-mediated cytotoxicity, cytokine signaling, necroptosis, and multiple amino acid metabolic processes, suggesting a more immune-engaged and metabolically diverse tumor environment (Figure 1D).

### 3.2. WGCNA Identifies Immune-Related Gene Modules Associated with Non-Metastatic PCa

**Figure 2:**
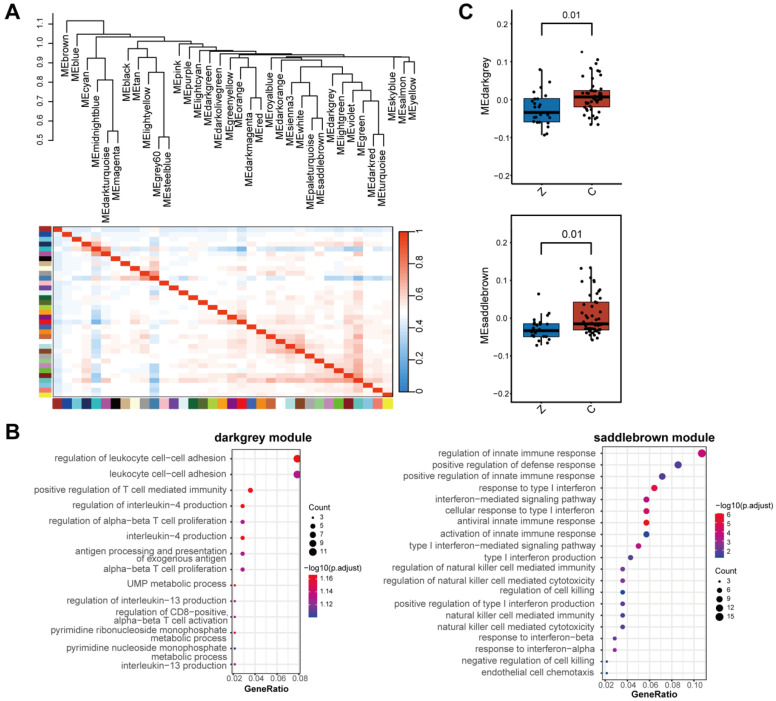
Identification of immune-related gene modules by WGCNA. (**A**) WGCNA dendrogram showing hierarchical clustering of genes and identification of 16 distinct co-expression modules, each represented by a unique color. (**B**) GO enrichment analysis of the darkgrey and saddlebrown modules. (**C**) Module-trait correlation analysis demonstrating the relationships between module eigengenes and clinical traits.

To investigate coordinated transcriptional programs linked to metastatic status, WGCNA was applied to the transcriptomic dataset. Sixteen distinct gene co-expression modules were identified, each represented by a unique color and reflecting specific biological programs (Figure 2A). Among these, enrichment analysis revealed that two modules were strongly associated with immune-related processes. The darkgrey module was primarily enriched for genes involved in IL4- and IL13-mediated signaling, as well as T cell proliferation and activation, implicating adaptive immune regulation (Figure 2B left panel). In contrast, the saddlebrown module was enriched for pathways related to interferon signaling, innate immune responses, NK cell-mediated cytotoxicity, and endothelial cell chemotaxis, indicating its involvement in innate immune surveillance and immune cell recruitment (Figure 2B right panel). Module-trait correlation analysis revealed that eigengene expression of both immune-related modules was significantly higher in non-metastatic tumors compared with bone-metastatic lesions (Figure 2C).

### 3.3. Immune Cell Deconvolution and Chemokine-Receptor Axis Analysis Reveal Endothelial-NK Cell Crosstalk in Non-Metastatic PCa

**Figure 3:**
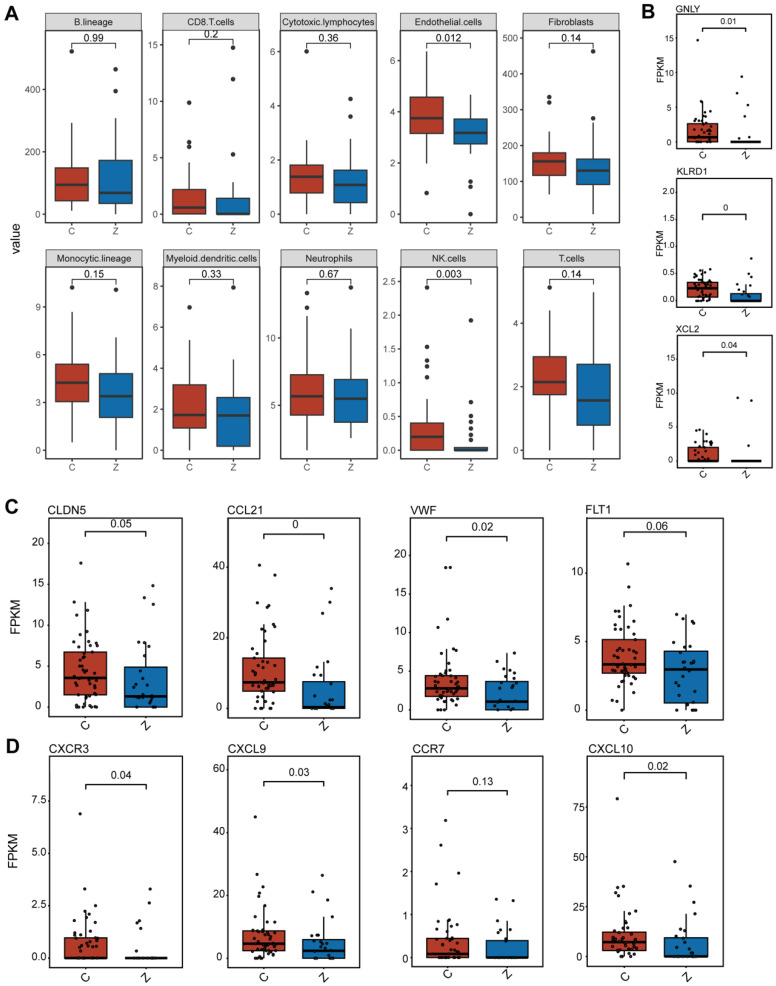
Immune landscape and chemokine profiling in non-metastatic and bone-metastatic prostate cancer. (**A**) MCPcounter analysis comparing the relative abundance of immune and stromal cell populations between non-metastatic and bone-metastatic PCa samples. (**B**) Differential expression of canonical NK cell markers between non-metastatic and bone-metastatic tumors. (**C**) Differential expression of endothelial cell-associated markers between the two groups. (**D**) Differential expression analysis of chemokines and chemokine receptors.

Given the immune-associated gene modules identified by WGCNA, we next characterized the immune and stromal landscape of PCa tissues using MCPcounter. This analysis revealed marked differences in tumor microenvironment composition between non-metastatic and bone-metastatic PCa. Notably, NK and endothelial cells were significantly enriched in non-metastatic tumors (Figure 3A). Consistently, canonical NK cell markers, including GNLY, KLRD1, and XCL2, were robustly upregulated in non-metastatic samples (Figure 3B), while endothelial-associated markers such as CCL21, CLDN5, and VWF also showed significantly higher expression (Figure 3C), indicating an immune- and vasculature-enriched microenvironment in localized disease. Given the concurrent enrichment of NK cells and endothelial cells, we further examined chemokine-receptor signaling pathways involved in immune cell recruitment. Transcriptomic analysis revealed that CXCR3 and its ligands CXCL9 and CXCL10 were significantly upregulated in non-metastatic prostate cancer tissues (Figure 3D). Based on these findings, we infer that endothelial cell may recruit and retain NK cells through chemokine-receptor interactions involving the CCL21-CCR7/CXCR3 and CXCL10-CXCR3 axes in non-metastatic PCa.

### 3.4. Single-Cell Transcriptomic Public Databases Validate Endothelial-NK Cell Crosstalk in Non-Metastatic Prostate Cancer

**Figure 4:**
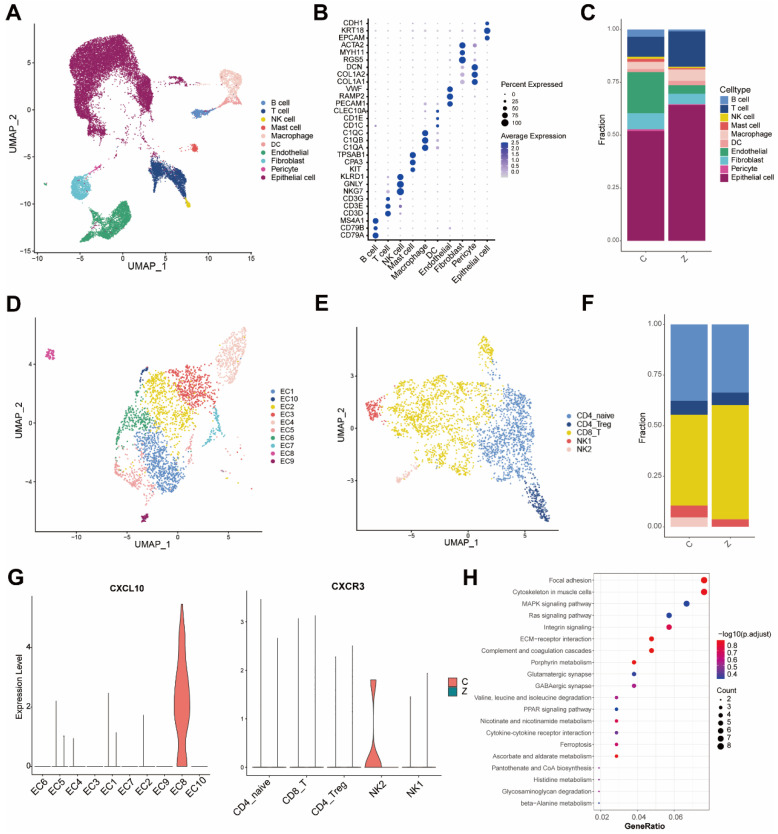
Analysis of the GSM4203181 dataset reveals immune microenvironmental differences between metastatic and non-metastatic prostate cancer. (**A**) UMAP visualization of major cell types annotated in the prostate cancer. (**B**) Dot plot showing the expression of canonical marker genes for each major cell type. (**C**) Bar plot depicting the proportional abundance of each major cell type across experimental groups. (**D**) UMAP visualization of endothelial cell subpopulations. (**E**) UMAP visualization of T and NK cell subpopulations. (**F**) Bar plot comparing the relative proportions of T and NK cells among groups. (**G**) Violin plots illustrating the expression levels of CXCL10 and CXCR3 across all identified cell subpopulations. (**H**) KEGG pathway enrichment analysis performed on the EC8 endothelial subcluster.

We utilized the publicly available single-cell RNA sequencing dataset GSM4203181 to validate the aforementioned findings. The dataset was generated using the 10 × Genomics 3′ Library and Gel Bead Kit V2. A total of nine samples were analyzed, including three bone metastatic samples (SC171, SC172, SC175) and six non-bone metastatic samples (SC155, SC156, SC159, SC162, SC173, SC174). The total number of cells obtained after quality control was 22,026. Following data quality control, batch-effect correction, and clustering, we identified ten major cell types based on established marker genes (Figure 4A,B): B cells (expressing CD79A, CD79B, and MS4A1), T cells (CD3D, CD3E, CD3G), NK cells (CD3^−^, NKG7, GNLY, KLRD1), macrophages (C1QA, C1QB, C1QC), Mast cells (KIT, CPA3, TPSAB1), dendritic cells (CD1C, CD1E, CLEC10A), endothelial cells (PECAM1, RAMP2, VWF), fibroblasts (COL1A1, COL1A2, DCN), pericytes (RGS5, MYH11, ACTA2), and epithelial cells (EPCAM, KRT18, CDH1). Consistent with our earlier observations, the non-metastatic group exhibited a higher proportion of NK cells and endothelial cells compared to the metastatic group. Further subclustering of endothelial and NK cells revealed ten endothelial subpopulations (Figure 4D), among which EC8 specifically expressed CXCL10 and was exclusively detected in the non-metastatic group (Figure 4G). Similarly, NK cells were subdivided into two clusters (Figure 4E), with CXCR3 being specifically expressed in the NK2 subset and only observed in non-metastatic samples (Figure 4G). These findings suggest a potential interaction between the EC8 subpopulation, via its ligand CXCL10, and the CXCR3 receptor on NK2 cells, which may facilitate the recruitment and retention of NK2 cells in the tumor microenvironment. KEGG pathway analysis further indicated that the EC8 subpopulation was significantly enriched in pathways related to ECM-receptor interaction and cytokine-cytokine receptor interaction. In summary, analysis of this public single-cell transcriptomic dataset corroborates our previous conclusions regarding endothelial-NK cell crosstalk in non-metastatic prostate cancer.

### 3.5. CXCL10-CXCR3 Co-Expression in TCGA-PRAD Associates with Immune Activation and NK Cell Signatures

**Figure 5:**
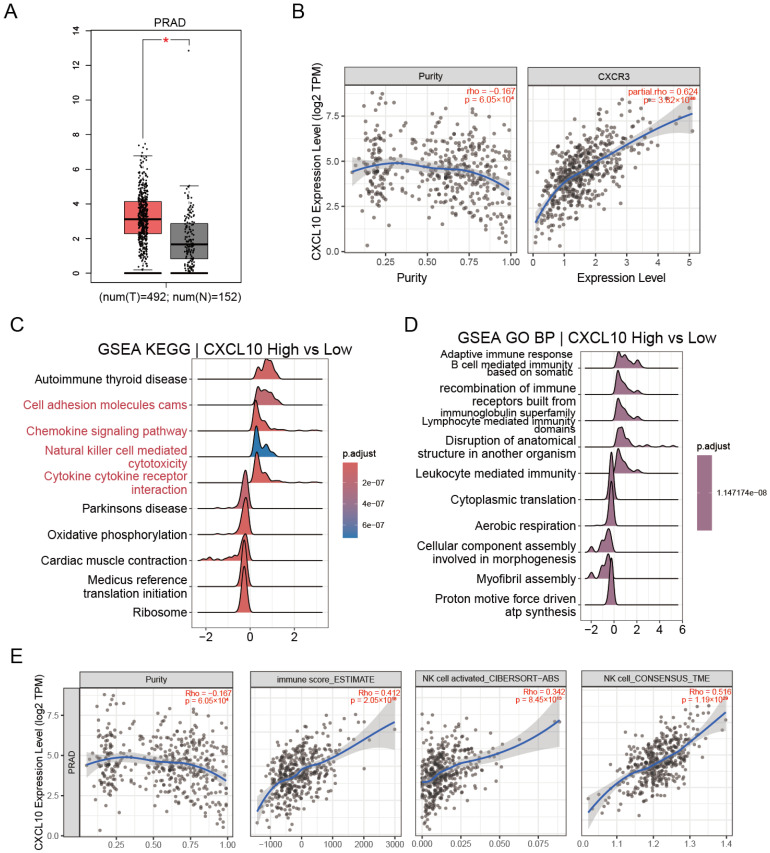
CXCL10 expression in the TCGA-PRAD cohort and its association with CXCR3, and NK-cell activation. (**A**) CXCL10 expression levels were compared between TCGA-PRAD tumor tissues and normal prostate tissues. * *p* < 0.05. (**B**) TIMER correlation analyses: correlation between CXCL10 and tumor purity (left); purity-adjusted partial Spearman correlation between CXCL10 and CXCR3 (right). (**C**,**D**) Samples were grouped by CXCL10 expression levels, and GSEA was performed by comparing CXCL10-high versus CXCL10-low tumors; enriched KEGG pathways (**C**) and GO Biological Process terms (**D**) are shown, with colors indicating multiple-testing adjusted *p* values (*p*.adjust/FDR). (**E**) TIMER immune infiltration analyses showing correlations between CXCL10 and ESTIMATE immune score as well as NK cell-related metrics (CIBERSORT-ABS activated NK cells and CONSENSUS_TME NK cells). Smoothed trend lines with confidence bands are displayed.

As shown in Figure 5A, within the TCGA-PRAD cohort, CXCL10 expression was significantly higher in prostate cancer tissues than in normal prostate tissues, indicating an overall upregulation of CXCL10 in PRAD. In Figure 5A,B, after adjustment for tumor purity, CXCL10 exhibited a strong positive correlation with CXCR3 (partial.rho = 0.624, *p* = 3.82 × 10^−46^). Further stratification by CXCL10 expression and comparison between CXCL10-high and CXCL10-low tumors revealed that GSEA results (Figure 5C,D) were markedly enriched for immune-related programs, particularly pathways involved in chemokine signaling, cytokine-cytokine receptor interaction, and NK cell-mediated cytotoxicity, while cell adhesion-related pathways were also enriched. Consistently, Figure 5E showed significant positive associations between CXCL10 and overall immune infiltration as well as NK cell-related metrics, including ESTIMATE immune score (rho = 0.412, *p* = 2.05 × 10^−18^), CIBERSORT-ABS activated NK cells (rho = 0.342, *p* = 8.45 × 10^−13^), and CONSENSUS_TME NK cells (rho = 0.516, *p* = 1.19 × 10^−29^), suggesting that CXCL10-high tumors exhibit a more immune-inflamed phenotype accompanied by NK-cell activation features.

### 3.6. In Vitro Characterization of Chemokine Receptor and Ligand Expression

**Figure 6:**
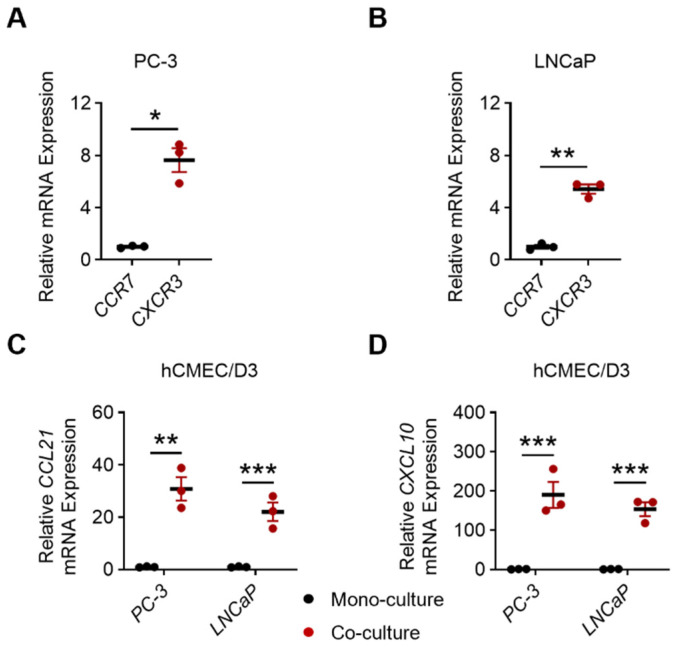
Validation of chemokine and chemokine receptor expression in vitro. (**A**) Relative mRNA expression levels of CCR7 and CXCR3 in PC-3 cells as determined by quantitative real-time PCR. (**B**) Relative mRNA expression levels of CCR7 and CXCR3 in LNCaP cells. (**C**) Induction of CCL21 expression in hCMEC/D3 endothelial cells following co-culture with PC-3 or LNCaP cells. (**D**) Induction of CXCL10 expression in hCMEC/D3 endothelial cells following co-culture with PC-3 or LNCaP cells. Data are presented as mean ± SD from three independent experiments. * *p* < 0.05, ** *p* < 0.01, *** *p* < 0.001 compared to control group by two-tailed Student’s *t*-test.

To determine whether PCa cells are capable of responding to chemokine signaling, we first assessed the expression of CCR7 and CXCR3 in PC-3 and LNCaP cell lines. qRT-PCR analysis demonstrated that both cell lines expressed CCR7 and CXCR3, with CXCR3 exhibiting higher basal expression levels compared with CCR7 (Figure 6A,B). We next investigated the regulation of chemokine ligand expression in endothelial cells. hCMEC/D3 were co-cultured with PC-3 or LNCaP cells, and chemokine expression was analyzed by qRT-PCR. Co-culture induced a marked upregulation of CCL21 (20–50-fold) and an even more pronounced increase in CXCL10 (150–200-fold) (Figure 6C,D), indicating strong induction of chemokine production through tumor-endothelial interactions.

### 3.7. CXCR3 Mediates CXCL10-Induced Survival Signaling in PCa Cells

**Figure 7:**
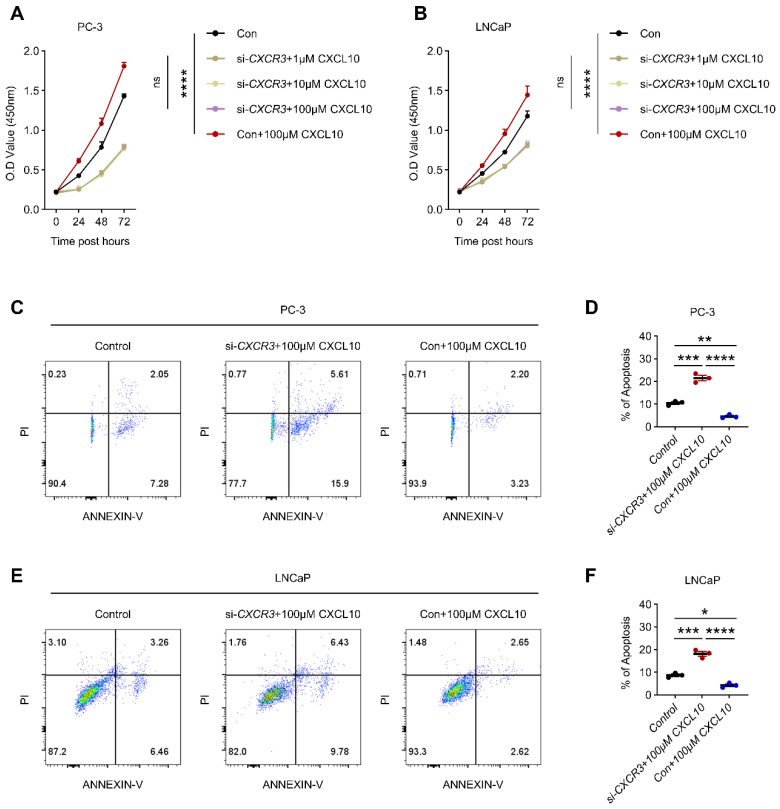
CXCR3 mediates CXCL10-induced survival and anti-apoptotic effects in prostate cancer cells. (**A**) Cell viability of PC-3 cells treated with increasing concentrations of CXCL10 or following siCXCR3 transfection, as assessed by CCK-8 assay. (**B**) Cell viability of LNCaP cells treated with increasing concentrations of CXCL10 or following siCXCR3 transfection, as assessed by CCK-8 assay. (**C**,**D**) Flow cytometric analysis of apoptosis in PC-3 cells treated with CXCL10 or transfected with siCXCR3. (**E**,**F**) Flow cytometric analysis of apoptosis in LNCaP cells treated with CXCL10 or transfected with siCXCR3. * *p* < 0.05, ** *p* < 0.01, *** *p* < 0.001, **** *p* < 0.0001.

Given the prominent expression of CXCR3 in PCa cells (Figure 6A,B), we next examined its functional role in mediating CXCL10-induced cellular responses. siRNA-mediated knockdown of CXCR3 was performed in PC-3 and LNCaP cells, and qRT-PCR and ELISA were confirmed a substantial reduction in CXCR3 expression compared with control cells, establishing effective suppression of receptor signaling (Supplementary Figure S1A–D). Functional assays demonstrated that CXCL10 treatment significantly enhanced cell viability in a dose- and time-dependent manner in control PC-3 and LNCaP cells, as assessed by CCK-8 assays (Figure 7A,B). In contrast, silencing CXCR3 markedly attenuated the growth-promoting effects of CXCL10 across multiple concentrations and time points (Figure 7A,B). Flow cytometric analysis further revealed that CXCL10 reduced apoptotic cell populations in control cells, consistent with its pro-survival effect (Figure 7C–F), whereas CXCR3 knockdown reversed this phenotype and significantly increased apoptosis despite CXCL10 stimulation (Figure 7C–F).

### 3.8. Endothelial-Derived CXCL10 Promotes PCa Cell Survival in Co-Culture Systems

**Figure 8:**
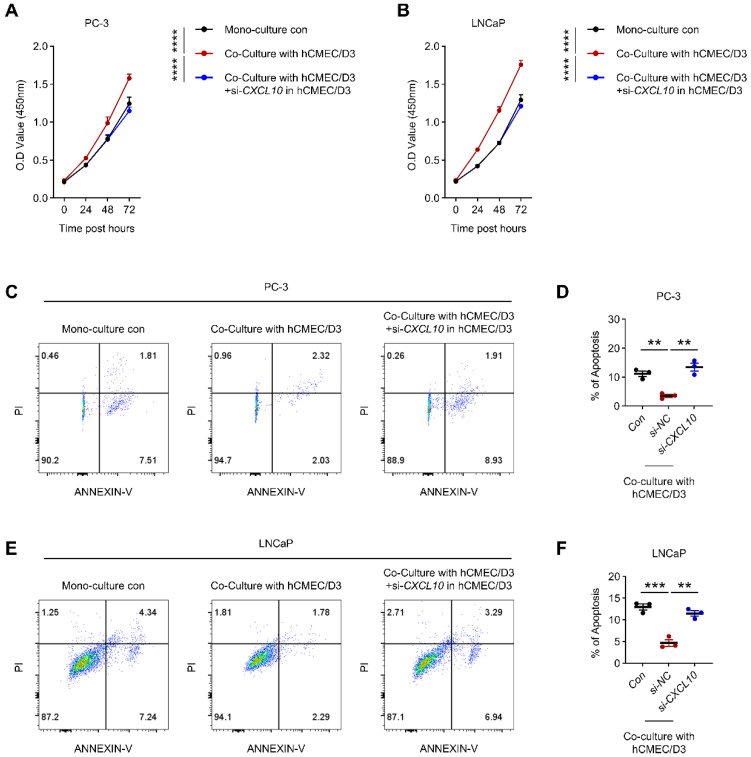
Effects of CXCL10 knockdown in hCMEC/D3 cells on the viability and apoptosis of prostate cancer cells. (**A**,**B**) Viability of PC-3 (**A**) and LNCaP (**B**) cells after co-culture with hCMEC/D3 cells transfected with siCXCL10 or siNC. (**C**,**D**) Apoptosis analysis of PC-3 cells under the same co-culture condition. (**C**) Representative flow cytometry plots. (**D**) Quantitative analysis of apoptotic cells. (**E**,**F**) Apoptosis analysis of LNCaP cells. (**E**) Representative flow cytometry plots. (**F**) Quantitative analysis. ** *p* < 0.01, *** *p* < 0.001, **** *p* < 0.0001.

To directly evaluate the contribution of endothelial-derived CXCL10 to PCa cell survival, siRNA targeting CXCL10 was introduced into hCMEC/D3 cells prior to co-culture with PC-3 or LNCaP cells. Efficient knockdown of CXCL10 expression in hCMEC/D3 cells was confirmed by RT-qPCR and ELISA (Supplementary Figure S2). In functional assays, co-culture with hCMEC/D3 cells significantly enhanced the viability of both PC-3 and LNCaP cells compared with monoculture conditions, indicating a strong survival-promoting effect mediated by endothelial cells (Figure 8A,B). Notably, this effect was largely abolished when CXCL10 expression was silenced in hCMEC/D3 cells, resulting in a marked reduction in cancer cell viability (Figure 8A,B). Consistently, flow cytometric analysis showed that endothelial co-culture significantly suppressed apoptosis in both PCa cell lines, whereas CXCL10 knockdown in hCMEC/D3 cells reversed this anti-apoptotic effect and increased apoptotic cell fractions (Figure 8C–F). Together, these findings demonstrate that endothelial-derived CXCL10 is a critical paracrine factor supporting PCa cell survival in the co-culture microenvironment.

### 3.9. CXCR3-CXCL10 Signaling Contributes to Docetaxel Resistance in PCa Cells

**Figure 9:**
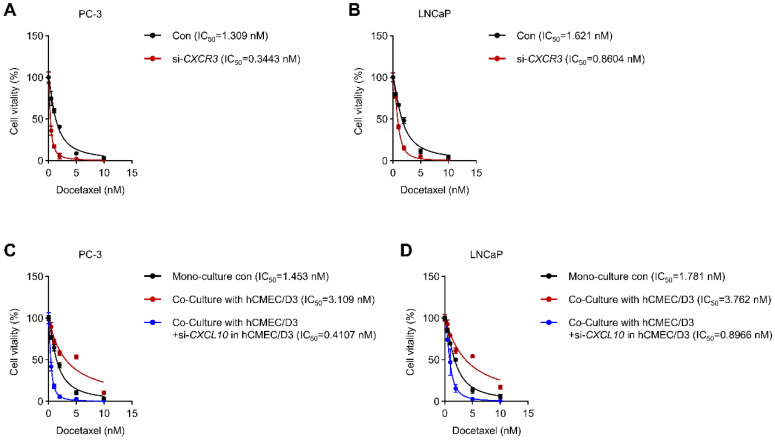
Effects of CXCL10/CXCR3 axis disruption on the sensitivity of prostate cancer cells to docetaxel. (**A**,**B**) Viability of PC-3 (**A**) and LNCaP (**B**) cells transfected with siCXCR3 or siNC and treated with increasing concentrations of docetaxel. (**C**,**D**) Viability of PC-3 (**C**) and LNCaP (**D**) cells treated with docetaxel after co-culture with hCMEC/D3 cells transfected with siCXCL10 or siNC.

We next investigated whether CXCR3-CXCL10 signaling influences the chemotherapeutic response of PCa cells. Dose-response analyses revealed that silencing CXCR3 significantly increased sensitivity to docetaxel in both PC-3 and LNCaP cells, as evidenced by pronounced reductions in IC_50_ values compared with control cells (Figure 9A,B). In contrast, co-culture with hCMEC/D3 cells markedly increased resistance to docetaxel in both PCa cell lines, resulting in elevated IC_50_ values relative to monoculture conditions (Figure 9C,D). Importantly, this endothelial-mediated chemoresistance was largely reversed when CXCL10 was knocked down in hCMEC/D3 cells, restoring docetaxel sensitivity to levels comparable to or lower than monoculture controls (Figure 9C,D). Collectively, these findings indicate that endothelial-derived CXCL10 promotes chemoresistance through CXCR3-dependent signaling, highlighting a functional role for tumor-endothelial crosstalk in modulating therapeutic response.

## 4. Discussion

**Figure 10:**
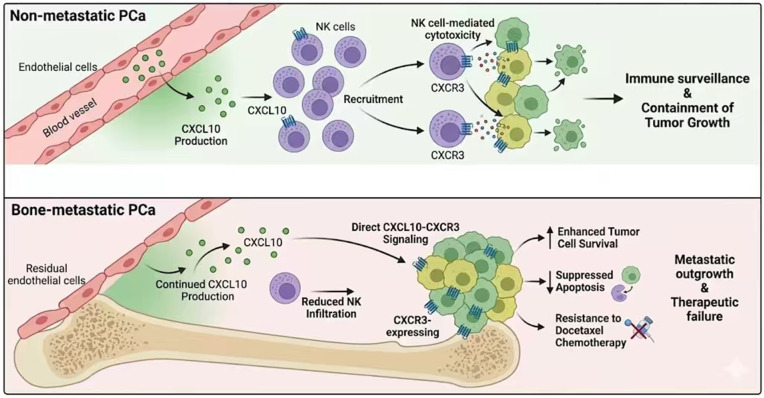
Schematic model illustrating the context-dependent dual role of the CXCL10-CXCR3 axis in prostate cancer progression.

Bone metastasis is a major cause of morbidity, therapeutic resistance, and mortality in advanced PCa, yet the mechanisms driving metastatic progression remain poorly defined. In this study, we performed transcriptomic profiling of 49 non-metastatic and 28 bone-metastatic PCa samples to systematically characterize molecular and microenvironmental differences associated with metastasis. WGCNA revealed that non-metastatic tumors are enriched for immune-related gene programs, which was further supported by immune deconvolution analysis demonstrating increased infiltration of NK cells and endothelial cells. Chemokine-cytokine axis analysis identified significant upregulation of CXCL10-CXCR3 signaling components in non-metastatic PCa. Importantly, in vitro functional experiments confirmed that CXCL10-CXCR3 signaling regulates PCa cell survival, apoptosis, and chemotherapeutic response. Together, these findings highlight a critical role for immune-associated chemokine signaling and tumor-microenvironment interactions in restraining PCa progression (Figure 10).

Our transcriptomic analyses revealed profound molecular differences between non-metastatic and bone-metastatic PCa. Bone-metastatic tumors were characterized by enrichment of ribosome-related pathways, suggesting enhanced translational activity, which is consistent with prior reports linking ribosomal biogenesis to aggressive tumor phenotypes and metastatic competence [16,17]. In contrast, non-metastatic tumors displayed enrichment of immune-related pathways, including NK cell-mediated cytotoxicity, cytokine signaling, and necroptosis. These findings indicate that immune surveillance is more active in localized disease and may be progressively attenuated during metastatic dissemination, supporting the concept of immune escape as a hallmark of metastatic PCa [18].

Through WGCNA, we further identified coordinated immune-related transcriptional programs that were preferentially active in non-metastatic tumors. Notably, both adaptive immune-associated modules (IL4/IL13 signaling and T cell activation) and innate immune modules (interferon signaling, NK cell cytotoxicity, and endothelial chemotaxis) were significantly downregulated in bone-metastatic disease. This coordinated suppression suggests that metastatic progression is accompanied not merely by loss of individual immune components, but by a global reprogramming of immune-stromal interactions within the tumor microenvironment [19]. Such coordinated immune attenuation has been reported in other metastatic malignancies and is increasingly recognized as a driver of immune evasion and therapeutic resistance [20].

Immune cell deconvolution further demonstrated that NK cells and endothelial cells were significantly enriched in non-metastatic PCa. NK cells play a critical role in controlling tumor dissemination and metastatic seeding through direct cytotoxicity and cytokine production [21]. The reduced NK cell infiltration observed in bone-metastatic tumors is therefore likely to contribute to metastatic outgrowth. Importantly, endothelial cells are not passive structural components but actively regulate immune cell trafficking through chemokine secretion and adhesion molecule expression [22]. The concurrent enrichment of endothelial cells and NK cells in non-metastatic tumors prompted us to explore chemokine-mediated endothelial-immune crosstalk as a potential regulatory mechanism.

Our chemokine-receptor axis analysis identified CCL21-CCR7/CXCR3 and CXCL10-CXCR3 as key signaling pathways enriched in non-metastatic tumors. CCL21 and CXCL10 are well-established endothelial-derived chemokines involved in immune cell recruitment, especially NK and effector T cells [23,24]. The marked upregulation of endothelial markers, together with increased chemokine expression and spatial proximity between NK cells and endothelial structures in non-metastatic tumors, supports a model in which endothelial cells actively orchestrate immune surveillance through chemokine gradients [25]. Loss of this chemokine network in bone-metastatic disease may therefore represent a critical step in immune exclusion.

Unexpectedly, our in vitro experiments revealed that endothelial-derived CXCL10 also exerts a direct tumor-promoting effect by enhancing PCa cell survival through CXCR3. While CXCL10 is traditionally regarded as an immune-attracting chemokine with anti-tumor functions, accumulating evidence indicates that CXCL10-CXCR3 signaling can be hijacked by tumor cells to promote proliferation, survival, and metastasis in a context-dependent manner [26,27,28]. We demonstrate that both PC-3 and LNCaP cells express CXCR3 at relatively high levels, and that CXCL10 stimulation enhances cell viability while suppressing apoptosis. Importantly, these effects are abolished upon CXCR3 knockdown, establishing a direct, receptor-dependent survival mechanism.

The dual role of CXCL10 revealed by our data highlights the context-dependent nature of chemokine signaling in the tumor microenvironment. This apparent paradox is consistent with emerging evidence that chemokines can exert opposing functions depending on the cellular context and disease stage [26,28]. In non-metastatic tumors, endothelial-derived CXCL10 may simultaneously promote NK cell recruitment and enhance tumor cell fitness, reflecting a dynamic equilibrium between immune surveillance and tumor adaptation [29,30]. However, as tumors progress to bone metastasis, immune evasion mechanisms progressively diminish NK cell infiltration, shifting the balance toward dominance of tumor-intrinsic CXCR3 signaling. In this context, CXCL10 produced by residual endothelial or stromal cells may be co-opted by tumor cells to directly support survival and confer chemoresistance [31,32]. This evolutionary model suggests that the net biological outcome of CXCL10-CXCR3 signaling depends on the relative abundance of CXCR3-expressing immune versus tumor cells within the microenvironment.

Our findings further demonstrate that endothelial-derived CXCL10 contributes to chemoresistance. Co-culture with endothelial cells significantly increased resistance of PCa cells to docetaxel, a standard therapy for advanced disease [33]. This resistance was largely reversed by CXCL10 knockdown in endothelial cells or CXCR3 silencing in tumor cells, indicating that CXCR3-CXCL10 signaling is a key mediator of microenvironment-induced drug resistance. Similar chemokine-driven resistance mechanisms have been reported in breast cancer and leukemia, where stromal-derived CXCL12 or CXCL10 activates survival pathways and attenuates chemotherapy-induced apoptosis [31,32,34].

Clinically, these findings have several important implications. Disruption of endothelial-immune crosstalk may represent a molecular hallmark of metastatic progression and could serve as a potential biomarker for identifying tumors with high metastatic propensity. In addition, CXCR3 expression on tumor cells delineates a subset of prostate cancers that appear to be particularly dependent on chemokine-mediated survival signaling within the tumor microenvironment [35]. From a therapeutic perspective, targeting CXCR3 signaling or endothelial-derived chemokine production may not only suppress tumor cell survival but also restore sensitivity to chemotherapy and potentially facilitate immune cell infiltration. Given the limited clinical benefit of immune checkpoint blockade in unselected prostate cancer populations [36], strategies aimed at modulating stromal-tumor communication and chemokine-driven signaling networks may provide a complementary avenue to remodel the tumor microenvironment and enhance therapeutic efficacy. Above all, our data resolve an apparent paradox by demonstrating that endothelial CXCL10 has a bifunctional role: it supports immune surveillance via NK recruitment, yet can be hijacked by CXCR3 high tumor cells to enhance fitness and drug tolerance.

Several limitations of this study should be acknowledged. First, although our transcriptomic and functional analyses reveal a strong association between the CXCL10-CXCR3 axis and prostate cancer progression, the retrospective nature of the clinical cohort limits direct causal inference regarding metastatic dissemination. Furthermore, all non-metastatic and bone-metastatic samples were obtained from different patients, and no matched primary tumor and bone metastatic tissues from the same individual were available for analysis. While such matched samples would provide valuable insights into the genomic evolution during metastatic progression, they are exceedingly difficult to obtain clinically. Second, while our study focused on endothelial-tumor crosstalk, future studies incorporating other microenvironmental cells such as macrophages, T cells, and osteoblasts will provide a more complete understanding of CXCL10-CXCR3 signaling in the bone metastatic niche. Third, while our in vitro co-culture models recapitulate key aspects of endothelial-tumor crosstalk, in vivo validation in orthotopic or bone metastasis models will be required to confirm the functional relevance of this axis in metastatic progression and therapeutic response. Finally, future investigations should explore the downstream signaling pathways activated by CXCR3 in prostate cancer cells and assess whether pharmacologic inhibition of this axis can synergize with chemotherapy or immunotherapy in preclinical and clinical settings.

## 5. Conclusions

In summary, our study reveals a context-dependent role of endothelial-derived CXCL10 in prostate cancer progression. While associated with immune-enriched microenvironments in non-metastatic disease, CXCL10-CXCR3 signaling directly promotes tumor cell survival and chemoresistance. These findings provide mechanistic insight into how tumor-endothelial interactions evolve during metastatic progression and identify the CXCR3-CXCL10 axis as a potential therapeutic vulnerability in advanced prostate cancer.

## Figures and Tables

**Table 1:** Clinicopathological characteristics of 77 prostate cancer patients.

Characteristic	Non-Metastatic (*n* = 49)	Bone-Metastatic (*n* = 28)
Age at surgery, median (range)	69 (48–82)	68 (51–80)
Preoperative PSA, ng/mL, median (range)	12.7 (1.83–183)	15.9 (4.5–155)
Biopsy Gleason score, *n* (%)		
≤6	12 (24.5%)	2 (7.1%)
7	24 (49.0%)	12 (42.9%)
8–10	13 (26.5%)	14 (50.0%)
Radical prostatectomy Gleason score, *n* (%)		
≤6	2 (4.1%)	0 (0%)
7	35 (71.4%)	15 (53.6%)
8–10	12 (24.5%)	13 (46.4%)
Pathological T stage, *n* (%)		
T2b	7 (14.3%)	2 (7.1%)
T2c	29 (59.2%)	12 (42.9%)
T3a	11 (22.4%)	8 (28.6%)
T3b	3 (6.1%)	5 (17.9%)
No record	0 (0.0%)	1 (3.6%)
Pathological N stage, *n* (%)		
N0	45 (91.8%)	21 (75.0%)
N1	4 (8.2%)	6 (21.4%)
No record	0 (0.0%)	1 (3.6%)
Bone metastasis at diagnosis, *n* (%)	0 (0.0%)	28 (100.0%)

## Data Availability

The raw data supporting the conclusions of this article will be made available by the authors on request.

## Supplementary Materials

The following supporting information can be downloaded at: https://www.mdpi.com/article/10.3390/biomedicines14040943/s1, Supplementary Figure S1. Validation of siRNA-mediated CXCR3 knockdown in prostate cancer cells. (A) qRT-PCR analysis confirming the knockdown efficiency of CXCR3 in PC-3 cells transfected with siCXCR3 compared with siNC control. (B) qRT-PCR analysis confirming the knockdown efficiency of CXCR3 in LNCaP cells transfected with siCXCR3 compared with siNC control. (C) ELISA analysis confirming the knockdown efficiency of CXCR3 in PC-3 cells transfected with siCXCR3 compared with siNC control. (D) ELISA analysis confirming the knockdown efficiency of CXCR3 in LNCaP cells transfected with siCXCR3 compared with siNC control. Data are presented as mean ± SD from three independent experiments. * *p* < 0.05, ** *p* < 0.01, *** *p* < 0.001 compared to control group by two-tailed Student’s *t*-test. Supplementary Figure S2. Validation of siRNA-mediated CXCL10 knockdown in endothelial cells. (A) qRT-PCR analysis of CXCL10 expression in hCMEC/D3 cells transfected with siCXCL10 or siNC following co-culture with PC-3 cells. (B) qRT-PCR analysis of CXCL10 expression in hCMEC/D3 cells transfected with siCXCL10 or siNC following co-culture with LNCaP cells. (C) ELISA analysis confirming CXCL10 expression in hCMEC/D3 cells transfected with siCXCL10 or siNC following co-culture with PC-3 cells. (D) qRT-PCR analysis confirming CXCL10 expression in hCMEC/D3 cells transfected with siCXCL10 or siNC following co-culture with LNCaP cells. * *p* < 0.05, ** *p* < 0.01, *** *p* < 0.001, **** *p* < 0.0001. Table S1. Clinicopathological characteristics of 77 prostate cancer patients.
