# Supplementary figures and images for: Single Cell and Bulk RNA-Seq Profiling of Non-Metastatic Versus Bone-Metastatic Prostate Cancer Identifies the CXCL10-CXCR3 Axis as a Key Determinant of Tumor Microenvironment and Treatment Resistance
Source: Biomedicines. 2026 Apr 21;14(4):943. doi: 10.3390/biomedicines14040943 (PMC13113686; doi:10.3390/biomedicines14040943)

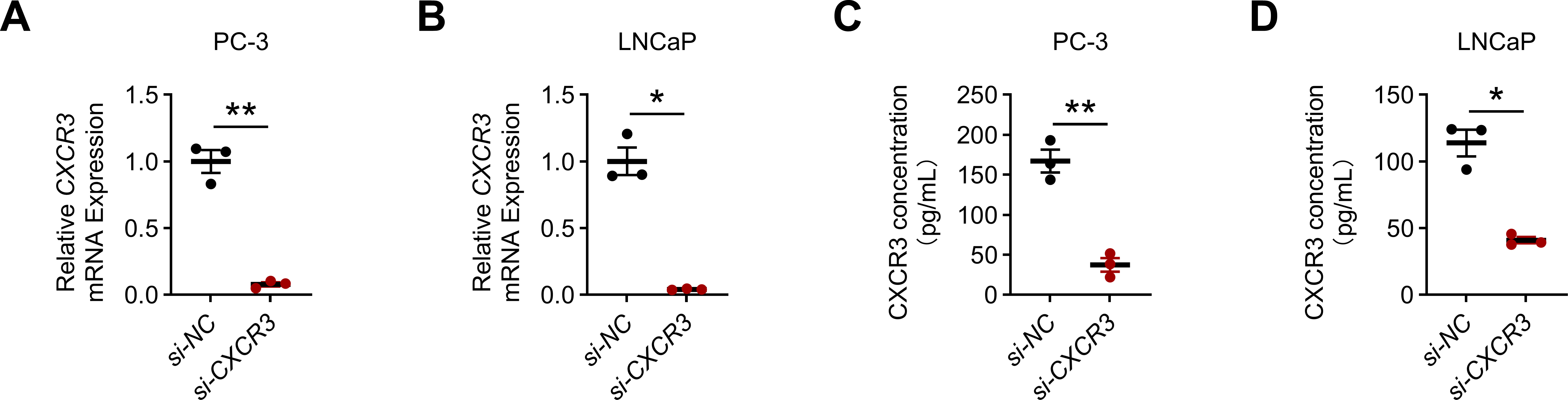

Supplement: Supplementary file 1 [file biomedicines-14-00943-s001.zip › Supplementary Figure S1.tif]

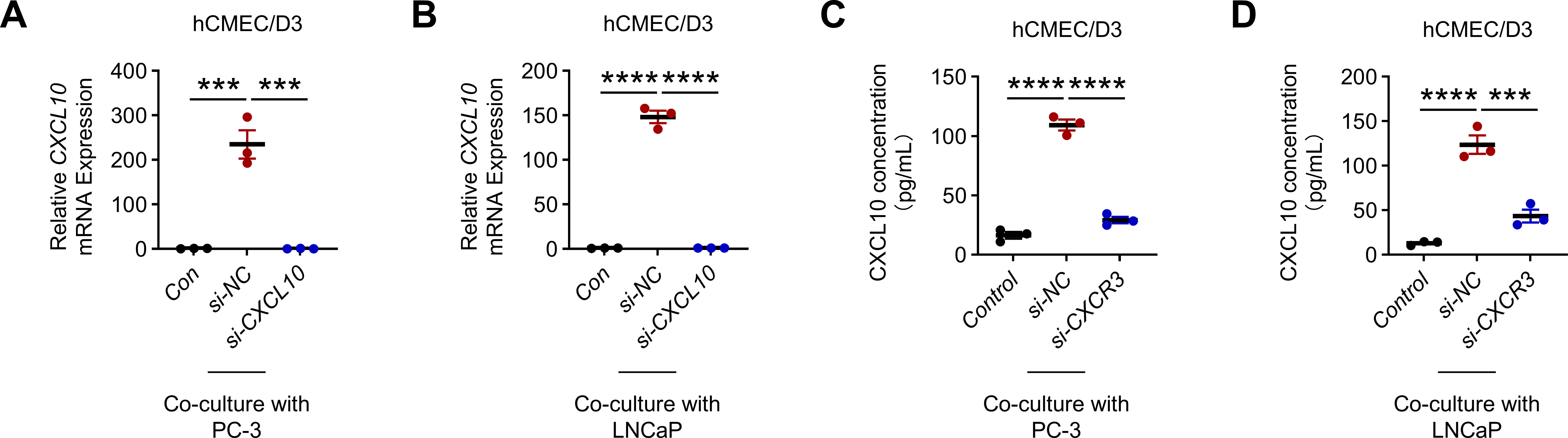

Supplement: Supplementary file 1 [file biomedicines-14-00943-s001.zip › Supplementary Figure S2.tif]
